# Adjusting the Residual Stress State in Wire Drawing Products via In-Process Modification of Tool Geometries

**DOI:** 10.3390/ma14092157

**Published:** 2021-04-23

**Authors:** Markus Baumann, René Selbmann, Matthias Milbrandt, Verena Kräusel, Markus Bergmann

**Affiliations:** 1 Institute for Machine Tools and Production Processes, Chemnitz University of Technology, D-09126 Chemnitz, Germany; verena.kraeusel@mb.tu-chemnitz.de; 2 Fraunhofer Institute for Machine Tools and Forming Technology IWU, D-09126 Chemnitz, Germany; rene.selbmann@iwu.fraunhofer.de (R.S.); matthias.milbrandt@iwu.fraunhofer.de (M.M.); markus.bergmann@iwu.fraunhofer.de (M.B.)

**Keywords:** wire-drawing, residual stress modification, FE simulation, residual stress measurements by X-ray diffraction

## Abstract

After conventional forming processes, the residual stress distribution in wires is frequently unfavorable for subsequent processes, such as bending operations. High tensile residual stresses typically occur near the wire surface and normally limit further processability of the material. Additional heat treatment operations or shot peening are often used to influence the residual stress distribution in the material after conventional manufacturing, which is time- and energy-consuming. This paper presents an approach for influencing the residual stress distribution by modifying the forming process, especially regarding die geometry. The aim is to reduce the resulting tensile stress levels near the surface. Specific forming elements are integrated into the dies to achieve this residual stress reduction. These modifications in the forming zone have a significant influence on process properties, such as plastic strain and deformation direction, but typically do not influence product geometry. This paper describes the theoretical approach and model setup, the FE simulation, and the results of the experimental tests. The characterization of the residual stress states in the specimen was carried out through X-ray diffraction using the sin^2^Ψ method.

## 1. Introduction

**Figure 1:**
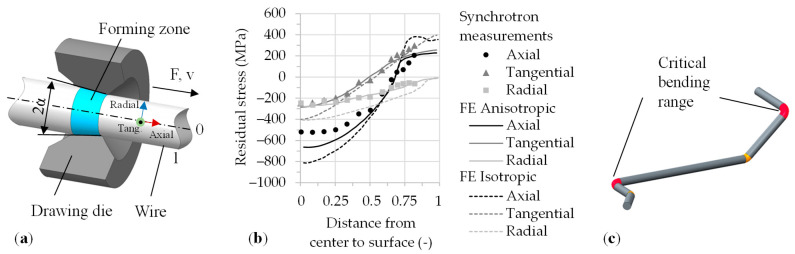
(**a**) Wire-drawing tool, (**b**) residual stress distribution to ref. [8], and (**c**) critical areas on a torsion bar spring.

The main production processes for elongated components comprise impact extrusion and drawing methods. One difference between these processes lies in the point of force application. In the forming zone, a combination of tensile and compressive stresses occurs. The cross-section is reduced during forming. Impact extrusion, as a press operation with applied compressive force, is preferably used for larger diameters and higher degrees of deformation. Wire forming with tensile force application is mostly used for smaller dimensions and smaller cross-sectional changes. Residual stresses occur in the material due to the elastic-plastic material behavior after removing all external forces. These stresses influence the subsequent forming operations of the semi-finished products and the mechanical application behavior of, e.g., the wire and semi-finished products [1]. Figure 1a shows a schematic structure of wire drawing. The arrow indicates the movement direction of the semi-finished product with the drawing speed and concurrent impact of the drawing force. The drawing tool is stationary and the geometry of the contour is cone-shaped, which is primarily determined by the taper angle (2α). The forming zone is located in the inlet cone region of the drawing die during wire drawing. The acting process force, triangle force, lateral force, and normal force arise corresponding to the taper angle. Additionally, the resulting vector between the normal force and the lateral force depends on friction. The forces cause axial, radial, and tangential stresses in the material. Corresponding to the force triangle, the deformation is predominantly affected by the normal force and resulting radial and tangential compressive stresses generated and, to a lesser extent, by the external drawing force [2]. Due to the elastic-plastic material behaviour, residual stresses occur in the wire after drawing. Residual stresses are mechanical stresses that result in a solid considered to be a complete system on which no external forces act [3]. Consequently, the balance of the internal forces is associated with the residual stresses [4]. These result from mechanically induced strain incompatibilities [5]. Resulting from the deformation during the wire-drawing process, the residual stresses (macroscopic distribution level averaged over several grains) are generated as tensile stresses near the surface and as compressive stresses in the core (Figure 1b). This characteristic residual stress distribution is unfavorable for subsequent forming operations, such as the bending of torsion bar springs as shown in Figure 1c. In the production process of torsion bar springs, these residual stresses specifically limit formability, as in bending operations with small bending radii in which the tensile residual stresses close to the surface add up to the load stresses of the bending deformation according to the superposition principle [6,7].

This stress distribution also tends to reduce the fatigue life of components. In order to counteract this residual stress distribution, a costly heat treatment is applied between the forming stages, or material depending on reshaping, or a second stage with less than 0.8% relative reduction in the cross-section [9]. The geometry of the tool has a great influence on the resulting residual stress state, therefore representing a suitable adjustment parameter for optimizing the residual stress distribution. Tekkaya [9] described and compared how the residual stress was influenced by changing the percentage reduction in the wire diameter in one conical die. Celentano et al. [10] demonstrated that an increase in the residual stresses was achieved by minimizing the drawing steps. Överstam [11], without using any special forming elements, investigated the influence of production tolerances and die geometry on the residual stresses. Atienza et al. [12] and Siva et al. [13] determined the modification of the taper angle and form of the die compared with the resulting residual stresses.

Overall, residual compressive stresses are preferable to residual tensile stresses in order to improve the fatigue strength. Residual compressive stresses cause delayed or suppressed crack growth. Llorca et al. [14] demonstrated that tensile residual stresses on the wire surface reduce the fatigue strength. However, an optimum result can only be achieved if the residual stress distribution corresponds to external forces or torque stress distribution.

One approach for influencing the residual stress distribution during the forming process lies in applying methods of gradation rolling and gradation extrusion, which have been developed to create materials with tailored properties [15,16]. Gradation extrusion appears to be particularly suitable, with additional severe plastic deformation (SPD) elements being integrated into the die. These elements create multiple local changes of the material flow along the surface contour of the tool. The forming process results in specific property modifications with gradients in microstructure and mechanical characteristics across the workpiece cross-section with property changes localized in the lateral area of the component [17,18]. Gradients of the micro and macro residual stresses are also expected to occur in the workpieces.

This work examined the mechanisms of gradation extrusion [16], particularly regarding the possibilities for influencing residual stress distributions due to the forming strategy for wire drawing processes. By influencing the stress distributions during forming of the semi-finished products, broader implications of subsequent processes are possible since additional processes, such as shot peening to adjust residual stresses, are not required. Reduced tensile or even compressive residual stresses after wire drawing in the near surface layer enable, e.g., smaller bending radii of torsion bar springs or increase the resistibility against cracks compared with conventionally manufactured products without any additional process steps to adjust the residual stress distribution. The presented results will be generalized and the subsequent mechanisms will be transferable to industrial wire drawing processes, such as basic manufacturing processes for semi-finished products of torsion bar springs. Subsequently applied bending operations are not considered here.

## 2. Materials and Methods

For the studies of the wire-drawing process with modified geometries of the drawing dies, single-phase ferrite steel S355 (DIN EN 10025-2: 2004) was used as the rod material with a diameter (D) of 12 mm. In order to achieve uniform initial conditions before forming, the material was annealed at 650 °C for 120 min and cooled in the oven after manufacturing of the specimens.

The cylindrical specimens consisted of a representative part (D = 12 mm, height = 100 mm), which was formed in the drawing experiments, and a part for the assembly to the drawing die and clamping in the tool (D = 9.6 mm, height = 225 mm). The transition zone between these parts was provided with a chamfer of 6° to generate a suitable inlet into the drawing die, similar to sharpening in industrial processes.

A solid lubricant (LOCTITE LB 8191) was applied to each of the specimens, and additional lubrication with high-alloyed drawing oil was used during forming. The experiments occurred at room temperature (20 °C). An experimental tool was constructed in order to investigate the mechanisms of generating residual stresses during wire-drawing processes. The tool consisted of column guides, a clamping unit for the specimens, interchangeable drawing dies and force, and position sensors. The tool was installed in a servo press and fixed to the press table and ram. The forming movement was carried out by the upward stroke of the press ram at a velocity of 20 mm/s. During the tests, the drawing force and movement were measured. 

The 0° position was marked on the specimens and the manufactured drawing dies were measured by tactile methods at the positions of 0°, 90°, 180°, and 270°. In the experiments, we determined that the specimens could be drawn reproducibly depending on their position in relation to the drawing die. Furthermore, the residual stress measurement could be assigned if the 0° markings of the specimen and the drawing die were matched in the experiment. In addition, the geometric dimensions of the drawing die, which were determined by tactile measurements, were also taken into account in the finite element (FE) simulation. 

**Figure 2:**
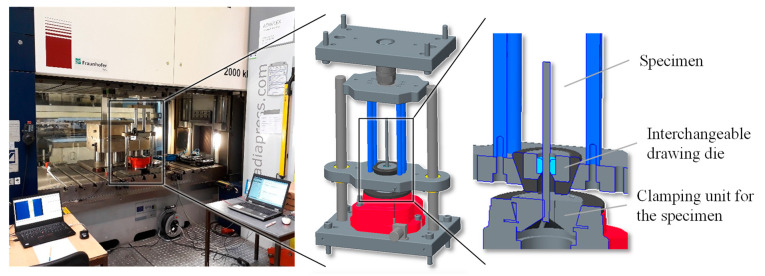
Tool principle—interchangeable dies for different geometry variants.

Figure 2 shows the tool design. For each investigated die geometry variant, three specimens were drawn through the die under constant conditions. 

**Figure 3:**
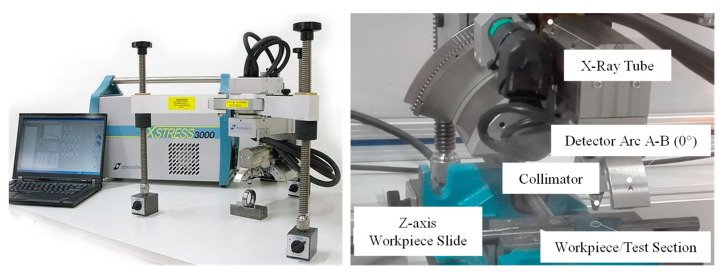
XRD analysis—basic system and measurement setup for wire workpiece.

The formed samples were analyzed by the X-ray diffraction method (XRD) and compared with the initial state. The residual stress states were determined in order to study the influence on the residual stress state by varying the tool geometry. The aim was to analyze correlations between the drawing die geometries and the resulting residual stresses after wire drawing. XRD allows for a phase-specific analysis of residual stresses, as the differences in the crystal lattices of the phases resulting from different Bragg angles and each phase can be analyzed by their reflections. In angle-dispersive X-ray diffraction, monochromatic X-ray radiation and Ω-2Θ mode were used for detecting ferrite-based residual stresses. The residual stress state was analyzed by the X-ray diffraction technique using the sin^2^Ψ method after calibrating the measuring system with a nearly stress-free ferrite calibration phantom. The ferrite-reflection 211 was selected for this analysis. Using Cr-Kα radiation (active wavelength 2.291 × 10^−10^ m), the reflection profiles were measured in the 2θ angular range of 148°–163°. The measurements were performed using a round collimator with a diameter of 2 mm. The reflection profiles were acquired for 11 Ψ tilts (±45°, ±39.2°, ±33.2°, ±26.6°, ±18.4°, and 0). An exposure time of 10 s in parallel with a collimator distance of 10 mm was used to determine the residual stresses. Moreover, measurement values were obtained by using a tube voltage of 30 kV and a tube current of 9 mA. For each wire-drawing variant and the initial state, three workpieces were measured identically at 0°, 90°, 180°, and 270° (12 sections) at the same position in axial and tangential directions. The surface of the wires was etched to a depth of 40 µm at the measuring point, which was in the middle of the drawn specimen length. Figure 3 illustrates the measurement setup.

**Figure 4:**
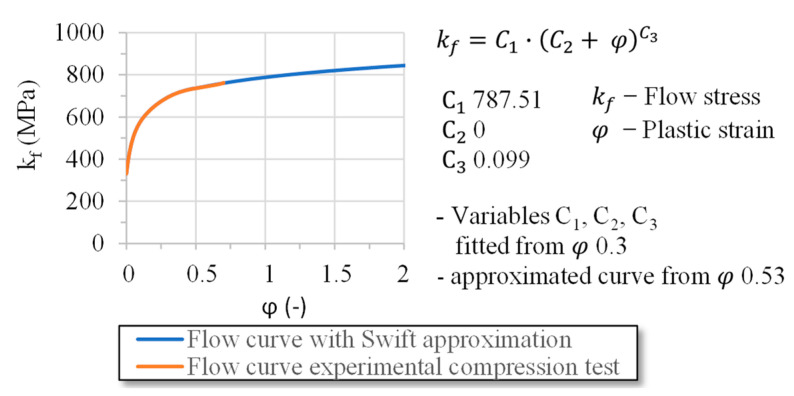
Flow curve S355 k_f_ (φ) for the FE simulations.

Flow curves were produced for material S355 for the FE simulation of the wire-drawing process. The specimens (D = 5 mm, height = 10 mm) were manufactured from the same rod material as the drawing specimens. The compression test’s strain rate of the three specimens was 0.1 1/s. The isotropic hardening behavior of the material was modeled using the Swift approach [19]. Figure 4 shows the flow curve and the extrapolation for higher plastic strain. The approximation was carried out using the plastic strain from φ 0.3 to the end of the measurement data. The parameters of C1 787.51, C2 0, and C3 0.099 were determined by applying the least squares method. Up to φ 0.53 the flow curve was represented with the measured values; for higher plastic strain, the extrapolation curve was used. 

**Figure 5:**
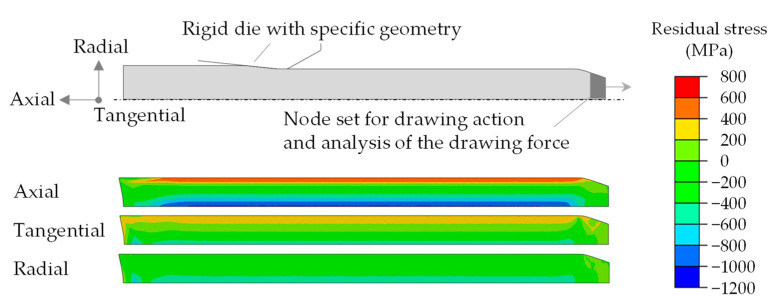
FE model setup and residual stresses after wire drawing (example conventional geometry).

In order to develop a basic selection of drawing dies and investigate the influence of the geometry elements on the resulting plastic strain, FE models were established in the Abaqus/CAE 2020 (Dassault Systèmes Simulia Corp., Johnston, RI, USA). For the first basic analyses of wire drawing, a 2D axially symmetric model structure was applied with an explicit calculation method. The specimens were defined in a simplified way with a residual stress-free initial state on the macroscale. To adjust the mesh during the forming process with an element edge length of 0.1 mm, the Abaqus method ALE Adaptive Mesh was used instead of remeshing. The friction factor model, a combination of the Coulomb and Tresca friction modeling, was applied. This friction model is used when the contact normal stresses are large, which the Coulomb model does not accurately represent [20]. The friction values were compared with the required drawing forces and adjusted (µ = 0.2, m = 0.2). The tools were modeled as rigid bodies. Following [16,21], the diameter of the starting material was reduced from D = 12 mm to D = 10.8 mm in one drawing step. To calculate the resulting residual stresses after forming and unloading, implicit calculations were performed. Figure 5 shows the 2D axially symmetric model setup with a rigid die. The drawing movement was generated via a node set, which was also used to evaluate the required drawing force. In the FE simulations, the specimens were drawn completely through the specific drawing dies. The kinematic contact method was used for surface contact condition. 

**Figure 6:**
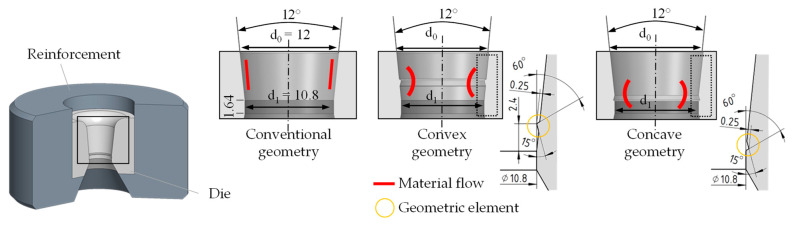
Geometry variants with special geometric elements.

When designing the dies, the initial aim was to introduce individual geometry elements and to uniformly implement the drawing angle 2α of 12° of the conventional die and the straight portion of the drawing die of 1.64 mm in the other variants. In addition to the conventional variant, the development included a convex or concave geometry element integrated into the drawing die. The geometric element of the convex and concave form had a size of 0.25 mm. The geometric elements incorporated in the forming zone caused plastic deformation during the forming process, leading to a change in the residual stress distribution of the material and affecting the material flow. However, the final diameter was not influenced. The angles and surfaces of the drawing dies’ inner contours were determined after production by means of tactile measurement and incorporated into the FE model as described above. Figure 6 illustrates the selected geometry variants as conventional, convex, and concave. 

## 3. Results

### 3.1. Results of FE Simulations

**Figure 7:**
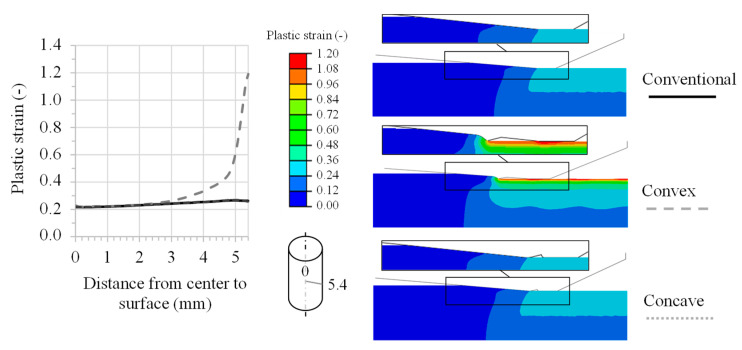
FE simulation of wire drawing—plastic strain over the specimen cross-section.

The analysis of the FE simulations shows that the convex element causes a significantly higher plastic strain on the specimen surface. Compared with the extrusion tests with the same die geometries [22], no increase in the plastic strain is achieved with the concave element due to the drawing force. The material flows over the concave element during the forming process without any significant change in the effective strain. Figure 7 illustrates the plastic strain over the cross-section. The die geometries were measured by tactile methods after production and integrated into the FE simulation. 

**Figure 8:**
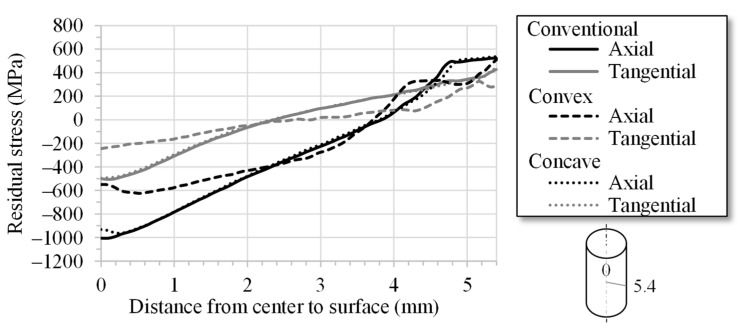
FE simulation of wire-drawing—axial and tangential residual stresses over the specimen cross-section as a function of the geometric elements.

The FE simulation analyses of the axial and tangential residual stresses illustrate that the residual stresses are influenced by the convex element over the entire cross-section profile due to the higher plastic strain. The radial component of the residual stress becomes zero at the surface and is not depicted. Figure 8 presents the curves for the axial (black) and the tangential (grey) residual stresses over the cross-section. The axial residual stresses have almost the same absolute value directly at the surface. However, below the surface, the residual stresses can be significantly reduced if the wire is drawn with the convex geometric element. In addition, the tangential residual stresses are smaller in the convex element compared with the conventional geometry. Due to the increased plastic strain in the peripheral zone with the application of the convex die geometry, the residual stress curve is influenced over the entire cross-section of the wire. The tensile residual stresses in the near-surface zone and the compressive stresses in the core are therefore reduced. 

### 3.2. Results of Experiments

**Figure 9:**
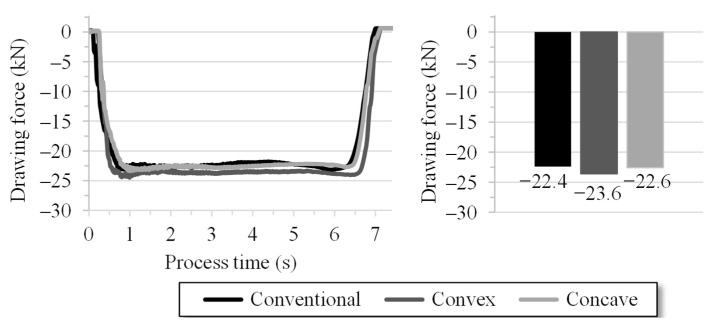
Experimental drawing forces of the die geometry variants, conventional, convex and concave (three specimens per variant).

Three specimens for each die variant were drawn through the die under constant conditions. The drawing process takes approx. seven seconds for the described setup. Figure 9 illustrates the average value for the drawing force curves. After a process initiation of one second, a continuous drawing process occurs up to the sixth second. This area is representative for the analysis. It shows that the drawing force for the conventional and concave geometries are almost identical. The drawing force required for the convex geometry is 5.3% above this value due to the higher plastic strain, which is depicted in Figure 7.

**Figure 10:**
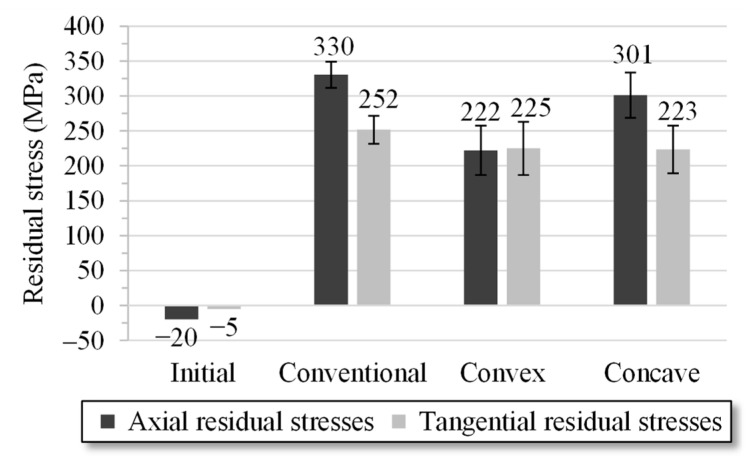
XRD-Measurements: Average residual stresses of the specimens drawn with conventional, convex, and concave die geometry variants (four measuring points on the circumference 0°, 90°, 180°, and 270°; three specimens per variant).

The axial and tangential residual stresses in the near-surface area were analyzed for the specimens using measurement methods as described in Section 2. Four measurement locations, located in the middle of the drawn area at 90° intervals around the circumference of the sample, were measured per specimen, equaling twelve measuring points for the three samples per geometry variant. The averaged results are shown in Figure 10.

The results show a reduction in the tensile residual stresses due to the specific die geometries compared with the conventional geometry. The residual stresses in the axial direction could be reduced by an average of 10% using the concave geometry. The tangential residual stresses were reduced to nearly 8%. If the wire was drawn with the convex die, the axial residual stresses reduced by as much as 30%. Compared with the FE simulations, differences in the absolute values still remained; the tendencies regarding wire drawing with the convex geometry element, however, indicated good correspondence, noting that the surface was etched by 40 µm. Differences occurred in the concave geometry. The FE simulation calculation was carried out using rigid dies. Therefore, one reason for the deviations between the experiments and the FE simulations may be minor changes in the elastic behavior of the die as a result of the process forces. In concave geometries particularly, small changes can have a major effect on the element in the form of higher plastic strain, implying a change in the residual stresses. The cylindrical guiding in the die can thus have a more significant influence when the wire experiences a minor relaxation at the concave element and returns to contact with the die after the element. This can be compared with a calibration, which here is more obvious than with the other variants. 

## 4. Discussion

Wire drawing was modeled using FE simulation and experiments. The experimental specimens were measured by XRD on the circumference. Differences in the residual stress values occurred at the surface between the variants that were drawn using conventional, convex, and concave die geometries. Using the convex geometry, a reduction in the tensile residual stresses of up to 30% was determined in the experiments compared with the conventional geometry. This reduction can be explained in connection with the increased plastic strain when using the convex geometry element. The higher plastic strain was also reflected in the increase in the drawing force. Deviations occurred in the absolute values of the FE simulations and the experiments, but the tendencies were evident. Therefore, residual stresses can be achieved with this process by applying individual elements in one drawing stage. Using this research approach, the properties of semi-finished products can be specifically adjusted by in-process modification of the drawing die geometries, which also implies the component properties in further manufacturing processes. 

## 5. Conclusions

Within the research activities, the forming process of wire drawing can be further investigated. Based on the FE simulation and the experimental results evaluated by the XRD measurements, the residual stresses can be characterized as an essential geometry-related feature for drawn wire samples. The residual stresses could be influenced by the specific application of individual small geometry elements in the drawing die’s forming zone. Based on these results, a modified wire drawing process can be further developed. The sequencing and combination of several elements in several drawing stages will be emphasized to implement the research results in a commercial wire-drawing process. Only in this manner can more favorable properties, particularly a reduced risk of cracking at very narrow bending radii, in the semi-finished wire also be reflected in the future component properties of wire products. In this context, the material model must also be expanded. The effects of the improved residual stresses have to be investigated and analyzed in specific bending experiments. In additional investigations, the surface properties after wire-drawing and the layers below the surface will be examined in relation to the geometry elements used.

## Data Availability

The data presented in this study are available on request from the corresponding author. The data are not publicly available due to privacy.
